# Systematic Review: Emotion Dysregulation and Challenging Behavior Interventions for Children andAdolescents with Autism with Graded Key Evidence-Based Strategy Recommendations

**DOI:** 10.21203/rs.3.rs-2802378/v1

**Published:** 2023-04-17

**Authors:** Heather J. Nuske, Amanda V. Young, Farzana Khan, Emma H. Palermo, Bukola Ajanaku, Melanie Pellecchia, Giacomo Vivanti, Carla A. Mazefsky, Lauren Brookman-Frazee, James C. McPartland, Matthew S. Goodwin, David S. Mandell

**Affiliations:** University of Pennsylvania; University of Pennsylvania; University of Pennsylvania; University of Pennsylvania; University of Pennsylvania; University of Pennsylvania; Drexel University; University of Pittsburgh; University of California, San Diego; Yale University; Northeastern University; University of Pennsylvania

**Keywords:** Emotion dysregulation, Challenging behavior, Externalizing symptoms, Evidence-based strategies, National Clearinghouse on Autism Evidence and Practice (NCAEP)

## Abstract

Challenging behavior, such as aggression, is highly prevalent in children and adolescents with autism and can have a devastating impact. Previous reviews of challenging behavior interventions did not include interventions targeting emotion dysregulation, a common cause of challenging behavior. We reviewed emotion dysregulation and challenging behavior interventions for preschoolers to adolescents to determine which evidence-based strategies have the most empirical support for reducing/preventing emotion dysregulation/challenging behavior. We reviewed 95 studies, including 29 group and 66 single-case designs. We excluded non-behavioral/psychosocial interventions and those targeting internalizing symptoms only. We applied a coding system to identify discrete strategies based on autism practice guidelines with the addition of strategies common in childhood mental health disorders, and an evidence grading system. Strategies with the highest quality evidence (multiple randomized controlled trials with low bias risk) were Parent-Implemented Intervention, Emotion Regulation Training, Reinforcement, Visual Supports, Cognitive Behavioral/Instructional Strategies and Antecedent-Based Interventions. Regarding outcomes, most studies included challenging behaviors measures while few included emotion dysregulation measures. This review highlights the importance of teaching emotion-regulation skills explicitly, positively reinforcing replacement/alternative behaviors, using visuals and metacognition, addressing stressors proactively, and involving parents. It also calls for more rigorously-designed studies and for including emotion dysregulation as an outcome/mediator in future trials.

## Introduction

**Figure 1:**
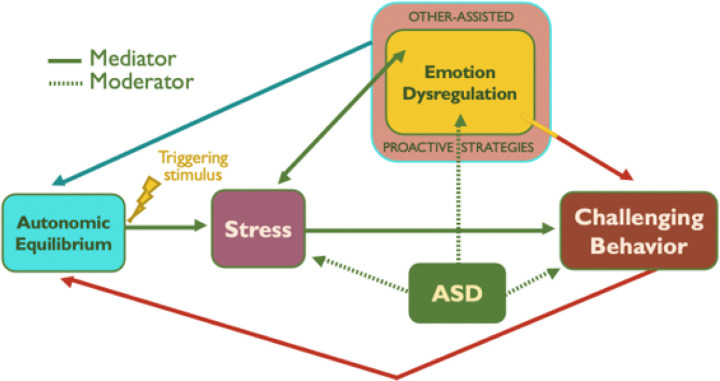
Transactional model of stress, emotion regulation & challenging behavior in autism spectrum disorder (ASD).

Challenging behavior – including aggression, self-injury, and property destruction – occur in as many as 80% of children and adolescents with autism spectrum disorder (ASD; [1] and can have a devastating impact. It negatively affects personal and family well-being [2] significantly impairs functioning [3], contributes to teacher burnout [4], and is the most frequent cause of hospitalizations among this population [5]. Although several reviews have examined strategies designed to address such behavioral challenges, no systematic review so far has focused on emotion dysregulation. Emotion dysregulation, i.e., difficulties with monitoring, evaluating, and modifying emotional responses to environmental demands [6] – is a common contributor to explosive outbursts and related challenging behaviors in ASD [7]. Individuals with ASD are more likely to experience stressful events compared to their peers due to their difficulties communicating, understanding others’ behavior and responding to demands, sensitivities to sensory stimuli, insistence on sameness, and social expectations that might be perceived as emotionally overwhelming [7]. A proposed theory is that the physiological and psychosocial stress frequently experienced by children and adolescents with ASD may culminate in challenging behaviors when they are unable to regulate their stress, i.e., when he or she does not have a repertoire of emotion regulation strategies to call on to defuse their stress [3]. Consistent with this notion, recent literature has documented that challenging behaviors in children and adolescents with ASD are more likely to surface in response to situations placing demands that exceed their emotion regulation skills [8]. This framework, whereby emotion dysregulation serves as a mediator between stress and challenging behaviors in ASD is illustrated in Fig. 1 [9].

The important role of emotion dysregulation in challenging behavior, externalizing behaviors, and outbursts has been well recognized in other populations [10,11]. Indeed, recommendations and practice guidelines for challenging behaviors across other disorders include evidence-based strategies to address emotion dysregulation [12] such as emotion regulation training, mindfulness-based interventions, and de-escalation training. However, these strategies have not yet been emphasized in major ASD intervention and practice guidelines for challenging behavior, despite their effectiveness in other populations. This may be because well-established ASD evidence practice guidelines on challenging behaviors are informed by literature reviews that do not include emotion dysregulation as a focus. The omission might reflect the epistemological and methodological tenets of behavioral disciplines [13], including Applied Behavior Analysis (ABA; [14], which has been most influential in the field of challenging behaviors in ASD and has historically focused on overt behavior as opposed to underlying “private events,” such as emotional processes (though we acknowledge this is not necessarily representative of modern ABA practices). This is in contrast with child mental health disciplines, for which emotion processing, reactivity and regulation are an important focus. Additionally, existing reviews on challenging behaviors have almost exclusively focused on single-case designs studies, which are most prominently used in the ABA field, and often exclude group-based study designs [15–17] which are more prominent in psychology/psychiatry fields, resulting in a limited focus on emotional dysregulation in conceptualizing challenging behavior and interventions to address behaviors in ASD.

To address this gap, the current systematic review builds and extends on the work by the National Clearinghouse on Autism Evidence and Practice (NCAEP) Evidence-Based Practices for Children, Youth, and Young Adults with Autism Spectrum Disorder 2020 Report [18], and previous reviews on challenging behavior interventions for children and adolescents with ASD [15–17,19]. The goal of the review is to extend previous literature search methods to provide a comprehensive re-examination and update on evidence supporting discrete strategies to reduce emotion dysregulation and challenging behavior in children and adolescents with ASD. We do this by addressing the limitations of previous reviews, including expanding search terms to include both challenging behavior and emotion dysregulation, including both single-case and group study designs to build upon the previous reviews in this area, and by considering interventions delivered across different settings, such as at school, home, clinic, and via telehealth.

We used the criteria for “evidence-based” as set by the NCAEP and cross-referencing an evidence grading system as outlined in Harbour and Miller [20] that rates studies by experimental control and methodological quality (e.g., risk of bias) in order to rank order evidence-based strategies based on empirical support. We use the term “evidence-based strategies” (instead of “evidence-based practices” used in NCAEP) to better align with the broader body of research on evidence based practices in child mental health disorders [21], as we are interested in discrete strategies rather than full intervention models.

## Methods

### Preregistration

Our search strategy was registered with International Prospective Register of Systematic Reviews (PROSPERO): ID [to be added after review]. This review’s pre-registered protocol is available here: [to be added after review].

### Search Strategy

**Figure 2:**
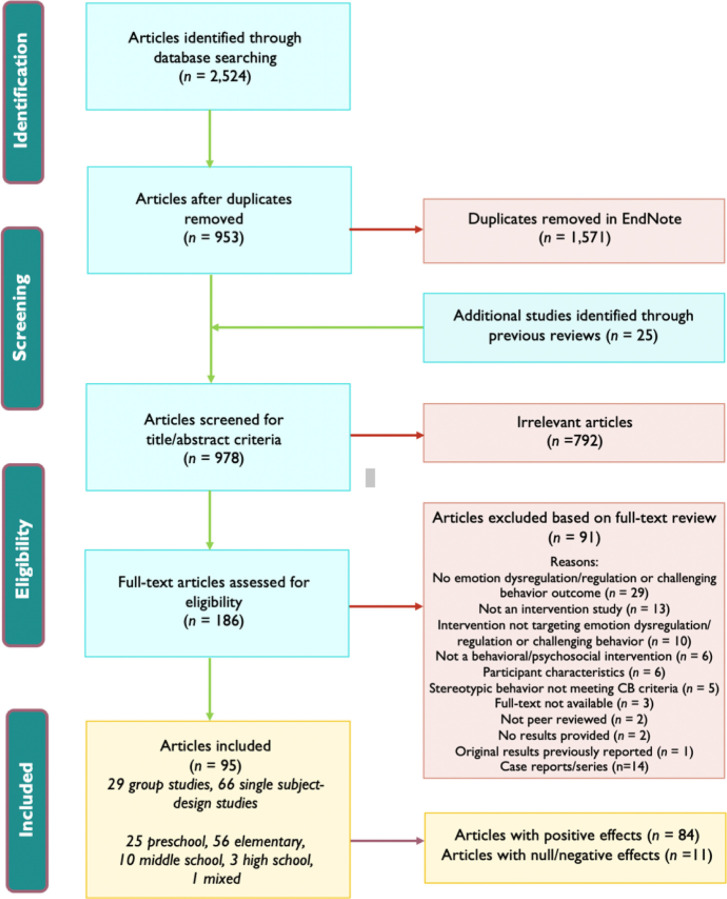
Article screening process and criteria.

Our team followed The PRISMA 2020 guidelines throughout this systematic review [22]. We identified studies through a systematic search of published articles on interventions for children and adolescents with ASD aimed at reducing challenging behavior or regulating emotions. We searched nine electronic databases based on the NCAEP 2020 Report [18]: (1) Academic Search Premier; (2) Cumulative Index to Nursing and Allied Health Literature (CINAHL); (3) Excerpta Medica Database (EMBASE); (4) Educational Resource Information Center (ERIC); (5) PsycINFO; (6) PubMed; (7) Social Work Abstract; (8) Sociology Abstracts; and (9) Web of Science. We used the following search terms: ab(emotion* regulation OR emotion* dysregulation OR self-regulation OR self regulation OR externaliz* OR challenging behavior* OR problem behavior* OR disruptive behavior* OR aggressi* OR irritab* OR coping skill* OR behavioral health) AND ab(Autis* OR autism spectrum disorder OR ASD OR ASC OR Asperger* OR Pervasive Developmental Disorder* OR PDD OR PDD-NOS) AND ab(intervention* OR strateg* OR trial* OR RCT OR program* OR approach* OR practice OR therap* OR treatment OR procedure OR method OR education OR curricul*) AND ab(school* OR home OR digital learning OR distance learning OR distance education OR remote learning OR remote education), modeling after and extending those included in the NCAEP 2020 Report [18] No child/adolescent focused terms or limits or limits on date of publication were included. The search was conducted on March 31, 2022. This initial search yielded 896 articles prior to eligibility coding. We found four previous reviews on challenging behavior interventions for children or adolescents with ASD and one chapter on emotion regulation interventions for children or adolescents with ASD [15–17,19,23], and we scanned their reference lists to determine if any other articles not previously included met our inclusion criteria. These reviews yielded an additional 25 articles prior to eligibility coding; therefore, our search yielded 921 articles to assess for eligibility. See Fig. 2 for article screening process and criteria used.

### Eligibility Criteria

The eligibility of each journal article was assessed for inclusion based on the following criteria: (1) the journal article was peer-reviewed; (2) it was written in (or translated into) English; (3) the study was specifically designed to target emotion regulation/dysregulation, externalizing behavior, or challenging behavior (i.e., behavior dangerous to the self or others, behavior that interferes with learning and development, and/or behavior that interferes with socialization or acceptance from peers); (4) the study included emotion dysregulation, emotion regulation, challenging behavior, and/or externalizing behavior as an outcome measure; (5) the children or adolescents were diagnosed with Autism Spectrum Disorder (ASD), Pervasive Developmental Disorder (PDD), or any subcategories of a PDD diagnosis; (6) the intervention evaluation design was either a group-based design (e.g., RCT, pre-post with/without a control group) or a single case design (e.g., multiple baseline, alternating treatment); and (7) the intervention was designed for school, home, clinic, or remote delivery. Exclusion criteria included: (1) medication only interventions; (2) interventions involving animals, massage, yoga, or physical therapy; (3) interventions only for adults; (4) interventions that targeted internalizing symptoms only; or (5) case reports/series. The role of emotion dysregulation in other psychopathology, such as internalizing behaviors, while recognized in the literature [11], was not included as it was outside of the focus of this review.

Initially, six raters assessed eligibility of the articles based on a review of abstracts. Abstracts were reviewed with a 20% overlap to assess interrater reliability (IRR = 79%; *κ* = .6), and all disagreements were reviewed with the team to reach a consensus on final determination per article. This yielded a total of 186 articles that were then included in a full-text review by four raters. All articles were double coded to determine if they met eligibility criteria (100% overlap). Raters reached high reliability (IRR = 84%; *κ* = .61). Again, all disagreements were reviewed with the team to reach a 100% consensus on final determination per article. See Appendix A for the full list of included studies.

### Methods and Design Data Extraction

The following information was extracted from each study: (1) author; (2) year; (3) country; (4) sample size; (5) sex; (6) age range; (7) IQ; (8) race/ethnicity; (9) delivery setting (and if school, school level, and classroom type); (10) intervention description; (11) delivery setting (school, home, clinic, or remote delivery); (12) teacher, therapist, parent, or research team mediated; (13) teacher/parent training methods; (14) control group description; (15) research design (quantitative, qualitative, mixed); (16) evaluation design (e.g., RCT, pre-post, multiple baseline, case study); (17) for single-case designs, number of repetitions/phases of intervention sequences; (18) for group designs, baseline group matching; and (19) reliability of primary outcome measures (e.g., Cronbach’s alpha, IRR, kappa).

### Evidence-Based Strategies Coding

We based our evidence-based strategy coding system on the Evidence-Based Practices for Children, Youth, and Young Adults with Autism Report (2020) by the NCAEP Review Team based at the University of North Carolina. Additional discrete strategies were considered for inclusion based on the criteria for qualifying as an evidence-based strategy outlined by the NCAEP Review Team: (1) two or more group design studies conducted by at least two different researchers or research groups; (2) five or more single case design studies conducted by three different investigators or research groups and with a total of at least 20 participants across studies; or (3) a combination of one group design and at least three single case study designs conducted by at least two different investigators or research groups. Following the NCAEP Review Team’s methods, we focused on discrete intervention strategies rather than comprehensive treatment models or full intervention packages therefore comprehensive treatment models or full intervention packages were coded on the discrete intervention strategies included in the model or intervention package. We considered adding the following additional discrete strategies (in alphabetical order; final list that met the above criteria provided in the Results section): Behavioral Descriptions/Reflections; Behavior Contracting; Behavior Management; De-Escalation; Emotion-Regulation Training; Exposure; Feedback; Homework; Metaphors/Analogies; Mindfulness-Based Strategies; Physical Safety Monitoring; Punishment; Role Play & Practice; Universal Behavior Management; Interpersonal Psychotherapy; and Verbal and Physical Comfort. See Appendix B for the full strategies coding system.

Evidence-based strategy coding was double-coded (i.e., 100% overlap) by two coders (lead coder a PhD ASD research psychologist), and two additional coders (one, a PhD school psychologist, Board Certified Behavior Analyst [BCBA] and ASD intervention researcher and trainer, and the other, a PhD clinical psychologist and ASD intervention researcher and trainer), completed a random selection of 20% of articles for reliability (IRR M = 92%, range 71–100%). If studies referenced previously peer-reviewed manuscripts in describing intervention components, those manuscripts were also reviewed for evidence-based strategy coding purposes. All disagreements were reviewed by the team to reach an ultimate consensus per study.

### Evidence Grade Coding

We used a coding scheme based on the evidence-based grading system outlined by Harbour & Miller (2001) [20] to weigh evidence per study design in a two-step process. First, numbered codes were applied per study: 1++ (RCTs with a very low risk of bias), 1+ (RCTs with a low risk of bias), 1− (RCTs with a high risk of bias), 2++ (high quality case-control or cohort studies with a very low risk of confounding, bias, or chance and a high probability that the relationship is causal), 2+ (well conducted case-control or cohort studies with a low risk of confounding, bias, or chance and a moderate probability that the relationship is causal) and 2− (case-control or cohort studies with a high risk of confounding, bias, or chance and a significant risk that the relationship is not causal). Studies rated 3 and 4 (non-analytic studies, case reports, case series, and expert opinions), were excluded as they were considered to carry the least scientific evidence based on Harbour and Miller’s evidence grade coding system. Extra specificity per grading level was taken from the Group Design Quality Appraisal Form and the Single Case Design Quality Appraisal Form from the Evidence-Based Practices for Children, Youth, and Young Adults with Autism Report (2020) by the NCAEP. See Appendix C for the full evidence grade coding system. All studies were independently double coded by two coders (IRR = 87% agreement) and discrepancies were reviewed to reach consensus. Ratings of individual studies included in this review have ranged from 1 + to 2−.

Second, based on Harbour & Miller (2001), [20] lettered codes were applied per evidence-based strategy based on a summary of the numbered ratings across studies: A (a body of evidence consisting principally of studies rated as 1 [i.e., RCTs] directly applicable to the target population and demonstrating overall consistency of results), B (a body of evidence including studies rated as 2++ [i.e., high quality single-subject design studies] directly applicable to the target population and demonstrating overall consistency of results), and C (a body of evidence including studies rated as 2 + directly applicable to the target population and demonstrating overall consistency of results). Therefore, the information extracted from this coding scheme indicates which evidence-based strategies have the strongest empirical support.

### Data Analysis

All extracted studies were considered when determining descriptive statistics on outcome measures (Table 1) but percentage of studies using reliable outcome measures were noted (for questionnaires and rating scales: Cronbach’s alpha/IRR ≥ .70, either reported in study or previously documented; for coding of challenging behavior: inter-rater reliability on 20% of observations [≥ 80% or ≥ .60 kappa]). Likewise, all extracted studies were considered when populating the initial list of discrete strategies for reducing emotion dysregulation/challenging behavior (Table 2), but strategies were rank-ordered by study design, i.e., group designs ranked higher than single-case study designs.

For the main analysis of evidence to support discrete strategies for reducing emotion dysregulation/challenging behavior, only the highest quality studies were included, including RCTs and high-quality single-subject design studies (rated 2++; see ‘Evidence Grade Coding’ section above and Appendix C), and the strategies were further broken down as A grade (tested in RCTs) and B grade (tested high quality single-subject design studies only).

## Results

### Study Characteristics

After assessing eligibility, a total of 95 articles were included in our review, which incorporated data from 2,092 children and adolescents with ASD across four continents (North America, Europe, Asia, and Oceania). These articles consisted of 29 group designs (15 RCTs, 7 with a non-randomized control group, and 7 without a control group), and 66 were single-case designs (42 multiple baseline/multiple probe, 12 withdrawal, 5 alternating treatments, 2 multiple-treatment, and 5 changing criterion). The number of studies per age of the individuals with ASD was as follows (mean or mode age used per study): 25 preschool (3–5 years), 56 elementary (6–11 years), 10 middle school (12–14 years), and 3 high school (15–18 years), 1 mixed (1 preschool student and 1 high school student). As would be expected for this population, most participants (82%) were male. Most studies did not include race/ethnicity (67%), but those that did showed 60% of participant were white, 18% Hispanic, 12% Black, 8% Asian, 2% multiracial, and 0.1% Native American. Likewise, most studies did not include an IQ score (74%), but across those that mentioned IQ or functional level, 55% of studies included children and adolescents with a below average IQ and/or who had significant support needs.

Regarding setting, 46% of studies implemented the strategies primarily at school (29% special education classroom, 9% general education/inclusion classroom, 14% mixed and 48% not specified), 29% primarily at home, 18% primarily through university or community clinics, and 5% primarily remotely via tele-health platforms. The percentage of studies in which the main person who mediated the strategies were teachers or teaching staff was 24%, parents was 15%, therapists was 9%, researchers was 21%, and 27% were split across person type (4% not specified). Training was provided to the child’s/adolescent’s educational or therapeutic team including parents in 80% of studies (20% of studies did not report training). Eighty-five studies (89%) found a positive effect on challenging behavior or emotion dysregulation outcomes (for single subject studies, on at least 80% of participants included), and 10 studies (10%) found a null/negative effect on outcomes. In studies with a positive effect on outcomes, 49/92 (53%) performed a Functional Behavior Assessment (FBA) to inform strategy selection, as opposed to 2/12 (17%) of null/negative effect studies. See Appendices D and E for methods details for each of the 95 included studies.

### Outcome Measures

As shown in Table 1, although most studies used live or video-tape coding of the frequency/severity/duration of challenging behavior as an outcome measure (71%), this was more common in studies using single-case relative to group designs. The next most common type of outcome measure was questionnaires or other rating measures of challenging behaviors frequency and/or intensity, followed by questionnaires or other rating measures of emotion dysregulation or self/emotion regulation. Most of the studies using these outcomes (≥ 80%) reported high reliability (criteria set same as in Appendix C: for questionnaires and rating scales, Cronbach’s alpha/IRR ≥ .70, and for coding of challenging behavior, interrater reliability on 20% of observations [≥ 80% or ≥ .60 kappa]). Few studies (10%) included an emotion dysregulation/regulation outcome measure, and those that did were more likely to be studies employing group designs. Only five (14%) group designs and zero single-subject designs included both challenging behavior and emotion dysregulation outcomes. No studies used emotion dysregulation/regulation as a mediator of outcome. See Appendix E for primary outcome measures of each study.

### Determination of “Evidence-Based” Strategies

Table 2 shows strategies evaluated against criteria based on those set forth by the NCAEP for evidence-based strategies (described in the Methods, above). Nine strategies were added based on their criteria (making 30 total): (1) Role Play and Practice; (2) Emotion Regulation Training; (3) Mindfulness-Based Strategies; (4) Homework; (5) Metaphors/Analogies; (6) Feedback; (7) Universal Behavior Management; (8) Physical Safety Management; and (9) Delay and Denial Tolerance Training. Of the top 10 in terms of empirical support, two (Role Play and Practice and Emotion Regulation Training) were not included in NCAEP nor other national guidelines in the United States (e.g., the National Standards Project). Appendix B provides a description of all strategies evaluated, broken into those meeting criteria and those not meeting criteria for an evidence-based strategy.

### Examination of Evidence-Based Strategies by Study Design, School Level and Delivery Setting

**Figure 3:**
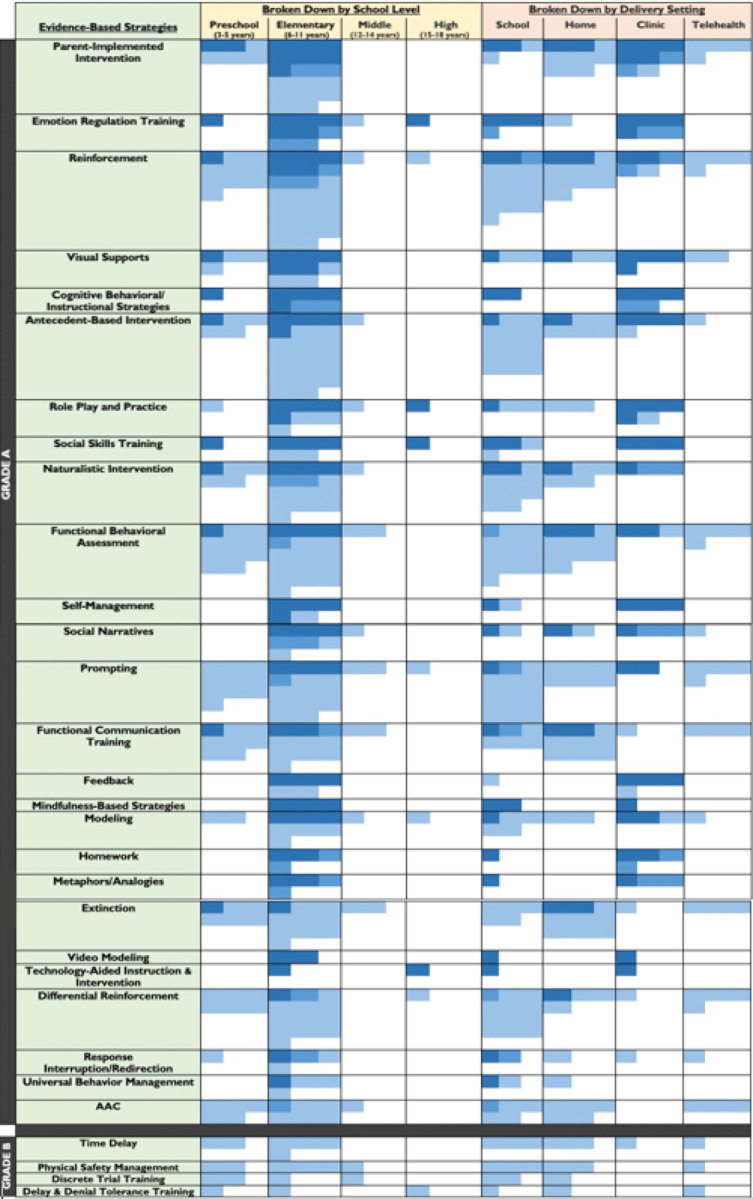
Evidence to support strategies for reducing emotion dysregulation/challenging behavior in high quality studies (only 1+, 1− and 2++ studies were included). Studies are broken down by school level and delivery setting. Each cell (blue/gray square) represents one study, and the shade of blue (gray) represents the quality of the study supporting the evidence-based strategy, with the darkest shade of blue (gray) representing 1+ studies (RCTs with low risk of bias), the medium blue (gray) representing 1− studies (RCTs with a higher risk of bias), and the lightest blue (gray) representing 2++ studies (high-quality case-control or cohort studies with a very low risk of confounding, bias, or chance and a high probability that the relationship is causal), as per Harbour & Miller (2001). Grade A strategies (indicated in the far-left column) include at least one RCT (i.e., studies rated 1+ and 1−) and Grade B studies do not (i.e., studies rated 2++). We do not include studies with null/negative findings (n=11), group studies with a control group but without all identified methods to reduce bias (rated as 2+, n=27), or group studies without a control group (rated as 2−, n= 6). See Appendix C for a full description on each evidence grade level and risks of bias assessed. In total 50 articles were included in this sub-analysis. School delivery was usually mediated through teachers, behavioral support professionals, one-to-one aides, or paraprofessionals. Home delivery was usually mediated via parents. See Appendices D and E for detail per study. AAC = Augmentative and Alternative Communication.

Figure 3 displays the empirical support (number and quality of studies) for each evidence-based strategy, rank ordered for evidence with quality. Only high-quality studies were included (RCTs and highest quality single-subject design studies; see Methods section ‘Evidence Grade Coding’ and Appendix C for evidence grading system; [20], including 50 studies (16 group designs and 34 single subject designs). To allow comparison of strategies empirically supported by randomized vs. non-randomized studies, strategies marked as “Grade A” include at least one RCT and the other strategies with only high-quality single-single design studies are marked as “Grade B”. Twenty-five of the evidence-based strategies met Grade A criteria and the remaining five met Grade B criteria [20].

The top ten evidence-based strategies rank ordered were Parent-Implemented Intervention, Emotion Regulation Training, Reinforcement, Visual Supports, Cognitive Behavioral/Instructional Strategies, Antecedent-Based Interventions, Role Play and Practice, Social Skills Training, Naturalistic Intervention and Functional Behavioral Assessment. Most (59%) of the evidence across all studies was for children with ASD in elementary school (6–11 years), few studies had a mean age in the middle (10%) or high school (3%) range. Only four strategies were tested in RCTs in high school students (Emotion Regulation Training, Role Play and Practice, Social Skills Training and Technology-Aided Instruction and Intervention), and *none* were tested in RCTs in middle-school students. Although the total number of studies were conducted in the school > home > clinic setting (rank order), the level of evidence across these settings was largely similar across contexts when considering study design. Studies on challenging behavior/emotion dysregulation interventions delivered via telehealth are emerging, with the most recent one being conducted out of necessity due to the COVID-19 pandemic [24].

## Discussion

This systematic review examined the evidence base on effective strategies to address emotional dysregulation and challenging behaviors in ASD. Unlike previous reviews, our focus encompasses both knowledge of evidence-based strategies to address challenging behaviors from the behavioral analytic field and knowledge on evidence-based strategies to support emotion dysregulation from the fields of psychology and psychiatry. We identified 30 evidence-based strategies that appear to have the most empirical support for addressing challenging behavior or/and emotion dysregulation, including 21 NCAEP identified strategies and nine ones that have been commonly used in the field childhood mental health but were unrecognized as part of the 2020 NCAEP report. Two of these previously unrecognized evidence-based strategies – Emotion Regulation Training, training to recognize one’s own bodily, cognitive, and behavioral signs of stress and employ proactive coping or emotion regulation skills to reduce one’s own stress, and Role Play and Practice, actively role playing with others to practice newly learned behavioral skills – were rated as a top 10 evidence-based strategy according to study design. This finding highlights the need to explicitly teach and actively engage children and adolescents with ASD in the recognition of their own emotional states and to help build their “toolbox” of emotion regulation strategies during times of distress, and to role play or practice other replacement behaviors.

Many of the strategies designated as evidence-based in this review targeting challenging behavior or emotion dysregulation do so indirectly through teaching replacement skills and strategies to reduce stress. The focus on skill building rather than punishing challenging behavior represents a departure from previously accepted practices (indeed Punishment was designated as a minimal evidence strategy) and reflects the importance of framing challenging behavior in the context of lagging skills [7,8]. At school, a behavioral episode may indicate that a child needs additional supports for example, if he/she/they demonstrate(s) escape behavior during a math lesson they may need visual supports to explain the math concept rather than a punishment, such as a timeout. By modifying the activity to support the individual the escape behavior will likely decrease. In the home environment, a child that hits their sibling because they want their toy may stop this behavior with parental prompting of requests for the toy from their sibling and positively reinforcing these communication attempts. Another example in the clinic setting, is that a child who self-harms may decrease this behavior through being taught how to recognize the early bodily signs of stress and use calming strategies such as sensory toys to address the child’s sensory needs. The key to addressing the lagging skills in each of these scenarios is to first work out the function that the challenging behavior has for the child.

Indeed, fourth highest on the evidence-based strategy list (Table 2) was FBA, a strategy classified with an evidence Grade A (Fig. 3), which is a widely recognized first step in intervening on challenging behavior in children and adolescents with ASD to determine the behavior’s function. For example, the Individuals with Disabilities Education Act (IDEA, 1997/2004) requires the use of FBA to develop plans targeting challenging behaviors and improving communication in individuals with disabilities. Although we listed FBA alongside the other strategies, it is best characterized as an assessment procedure for developing a behavior intervention plan, rather than an “intervention” strategy itself. Previous reviews differ in terms of characterizing FBA as a strategy (see different approaches by the NCAEP and the National Standards Project) and previous reviews on challenging behavior in ASD have drawn different conclusions regarding the central importance of FBAs. Some suggest it is not a necessary step as effectiveness does not seem to relate to completion of an FBA [16]. Others, however, suggest better outcomes relate to interventions that include FBA [15,17]. We found that studies with a positive effect on outcomes were more likely to have done an FBA than studies with null/negative effects on outcomes, which is suggestive of the critical importance of determining a behavior’s function before choosing which evidence-based strategy to implement. However important, performing FBAs in the school setting are often complicated by lack of professionals able to assess the child as many schools rely on external behavioral consultants to conduct these assessments. Given the critical importance of determining a behavior’s function to create successful behavioral plans, these results highlight the need for a new implementation approach for conducting FBAs in schools. For example, including professional development and coaching of teachers and other school staff on the data collection and/or analysis of FBAs, which could build on their foundational skills and training in behavioral data collection, could help to avoid the bottle neck that this can cause for developing and actioning behavior plans.

Relatively few studies incorporated emotion dysregulation or emotion regulation outcome measures, and these tended to be more recent studies (since 2015), with two exemptions, [25,26] marking that as a field there is growing emphasis on emotion dysregulation in ASD [7]. No studies incorporated physiological stress outcomes. Given that individuals with ASD often have difficulties expressing their emotions and stress [27–29], such a multimodal assessment approach could help triangulate findings or reveal insights into their behavior and response to intervention [23]. Studies using a transdisciplinary approach to examine both challenging behavior and emotion dysregulation outcomes are still emerging [25,26,30–33]. Future research in the area should aim to incorporate multiple assessment methods and outcome measures.

This review also highlights major gaps in the field in terms of studies covering certain developmental stages, contexts, and groups. Relatively few high-quality studies examined interventions targeting middle or high school students with ASD (though we acknowledge there are ongoing studies targeting this age range [34]; only four strategies were tested in RCTs in high school students, and none were tested in RCTs in middle-school students. There is a milieu of hormonal, social, and emotional changes that take place throughout middle childhood and adolescence, including new challenges and opportunities for experiencing and regulating emotions (or dysregulating) emotions, which may manifest as challenging behavior [35]. Therefore, it is important to consider only the specific evidence-based strategies tested in this age range as evidence-based for this age range, given that most participants included in the review were preschool or elementary school students. These findings highlight the clear need for future research to address this knowledge gap.

Moreover, our review highlighted the lack of female study participants and a lack of interventions delivered via telehealth, though we may expect more of the latter to come given the ongoing COVID-19 pandemic. Of note, a significant limitation was the inclusion of predominantly white samples wherever race/ethnicity was indicated in studies. This severely limits the ability to apply the results on what is considered evidence-based on a population-based level, since emotional dysregulation and challenging behavior may have deeper cultural biases and expectations (e.g., what is considered emotion dysregulation or challenging behavior). Future research should aim to include more racial and ethnic diversity in samples to determine if results on evidence-based strategies and considerations around choosing target behaviors generalize across racial/ethnic groups. Overall, these findings suggest the knowledge gained to date about evidence-based strategies to support children and adolescents with ASD in reducing emotion dysregulation and challenging behavior is representative only of a sub-group of younger, less racially/ethnically diverse, mostly male with ASD, but not necessarily applicable to the wider ASD population. Further research is needed to fill each of these crucial gaps.

Interestingly, we found that the proportion of null/negative effects was much lower in single-case design studies compared to group design studies, suggesting there may be publishing bias at play. Authors may choose to publish cases that have a higher rate of success in treatment, so attaining an accurate picture of null/negative effects using single-case design studies may be difficult [36]. This points to the importance of routinely reporting null/negative effects to allow for a more comprehensive and unbiased examination of the literature.

### Limitations

This systematic review is not without limitations. First, although the evidence level across all evidence-based strategies within each study was given equal weight in our review, some evidence-based strategies may have been more important than others (the “active ingredients”) in producing positive intervention effects. However, no studies examined discrete evidence-based strategies within intervention packages separately. This, and the use of outcome measures that are not comparable across studies, precludes a fine-grained analysis of specific components within different intervention packages. Examining active ingredients of challenging behavior/emotion dysregulation interventions in children and adolescents with ASD is an important future direction for the field. Additionally, for consistency, we limited coding of the discrete strategies to the articles, or if studies referenced previously peer-reviewed manuscripts in describing intervention components, those manuscripts were also reviewed. However, for intervention packages, this may mean that we missed discrete strategies that are in intervention manuals but not described in the peer-reviewed publications. Finally, our search criteria may have presented biases as grey/unpublished literature and articles not translated to English were excluded. As such, our study is not exempt from publication bias or selection bias as relevant articles (with both positive and null/negative effects) may have been missed.

## Conclusions

This systematic review synthesizes knowledge of evidence-based strategies to address challenging behavior in ASD from the behavioral analytic field with the knowledge of evidence-based strategies to support emotion dysregulation from the fields of psychology and psychiatry. This review highlights the importance of teaching emotion-regulation skills to children and adolescents with ASD explicitly, positively reinforcing their replacement/alternative behaviors, using visuals and metacognition to teach them skills, addressing their stressors proactively, and involving their parents. Our hope is that the findings of the current systematic review will facilitate further development, demonstration, and dissemination of evidence-based practices to support children and adolescents with ASD who engage in challenging behaviors more equitably and effectively.

## Figures and Tables

**Table 1:** Outcome Measures Across All Studies (n = 95)

Outcome Measure Type	Per Design		Total	Reliable Outcome Measures* N/Total (%)
			N/95 (%)	
	Group N/29 (%)	Single-Case N/66 (%)		
Observed frequency/severity/duration of challenging behavior	7 (24%)	62 (94%)	69 (72%)	56/69 (82%)
Observed challenging behavior reduction or time before challenging behavior onset	0 (0%)	2 (3%)	2 (2%)	1/2 (50%)
Observed frequency/severity/duration of emotion dysregulation or self/emotion regulation	1 (3%)	1 (2%)	2 (2%)	1/2 (50%)
Questionnaire/rating of challenging behaviors frequency and/or intensity	20 (69%)	1 (2%)	21 (22%)	19/21 (90%)
Questionnaire/rating of emotion dysregulation or self/emotion regulation	10 (34%)	0 (0%)	10 (11 %)	8/10 (80%)
Intervention steps (desensitization) successfully completed	0 (0%)	1 (2%)	1 (1 %)	0/1 (0%)
Anecdotal description of the challenging behavior	1 (3%)	1 (2%)	2 (2%)	N/A

* Number and percentage of studies per outcome measure type which included adequately reliable outcome measures (same criteria as for evidence ratings, Appendix C); for questionnaires and rating scales: Cronbach’s alpha/IRR ≥ .70, either reported in study or previously documented; for coding of challenging behavior: inter-rater reliability on 20% of observations (≥ 80% or ≥ .60 kappa)

**Table 2:** Discrete Strategies for Reducing Emotion Dysregulation/Challenging Behavior, Number of Studies and Inclusion in United States National Guidelines, Rank-Ordered by Design of Positive Effect Studies

Strategy	Positive Effect		Null/Negative Effect		# Research Groups (1, 2 or ≥3)	Included in NCAEP	Included in NSP
	# Group Designs	# Single-Case Designs	# Group Designs	# Single-Case Designs			
Reinforcement	14	45	4	3	≥3	✓	✓
Parent Implemented Intervention	14	15	1	1	≥3	✓	✓
Visual Supports	13	11	2	1	≥3	✓	✓
Functional Behavioral Assessment	10	41	2	2	≥3	✓	
Naturalistic Intervention	10	19	4	0	≥3	✓	✓
Emotion Regulation Training	10	9	1	1	≥3		
Cognitive Behavioral/Instructional Strategies	9	2	1	0	≥3	✓	✓
Prompting	8	37	0	3	≥3	✓	✓
Antecedent-Based Intervention	8	28	3	2	≥3		
Extinction	8	17	2	0	≥3	✓	✓
Role Play and Practice	8	12	0	0	≥3		
Mindfulness-Based Strategies	8	1	2	0	≥3		
Homework	8	0	1	0	≥3		
Functional Communication Training	7	22	1	1	≥3	✓	
Differential Reinforcement	6	29	3	2	≥3	✓	✓
Social Narratives	6	6	0	1	≥3	✓	✓
Augmentative and Alternative Communication	5	11	0	1	≥3	✓	
Self-Management	5	6	1	0	≥3	✓	✓
Modeling	4	11	0	2	≥3	✓	✓
Social Skills Training	4	7	4	0	≥3	✓	✓
Technology-Aided Instruction and Intervention	4	1	1	0	≥3	✓	
Metaphors/Analogies	4	0	1	0	≥3		
Feedback	3	6	0	1	≥3		
Discrete Trial Training	3	5	1	0	≥3	✓	✓
Response Interruption and Redirection	2	8	0	1	≥3	✓	✓
Universal Behavior Management	2	4	1	1	≥3		
Video Modeling	2	0	0	0	2	✓	✓
Physical Safety Management	0	9	1	2	≥3		✓
Delay and Denial Tolerance Training	0	7	0	0	≥3	✓	
Peer-Based Instructional Intervention	1	4	0	0	≥3	✓	✓
Exercise and Movement	1	1	1	0	≥3	✓	
Exposure	0	4	0	0	≥3		
De-Escalation	0	2	0	1	≥3		
Verbal and Physical Comfort	0	2	0	0	2		
Behavioral Movement Intervention	0	1	0	0	1	✓	
Punishment	0	1	0	0	1		
Behavioral Descriptions/Reflections	0	0	0	0	0		
Behavior Contracting	0	0	0	0	0		
Sensory Integration	0	0	0	0	0	✓	

*Note*.. Grayed out strategies are those with minimal evidence. See Appendix B for a description of each strategy. NCAEP = National Clearinghouse on Autism Evidence and Practice, NSP = National Standards Project

## Data Availability

All data, coding schemes and search syntax are available either in the manuscript or in the supplementary information.
